# The Phylogenomic Approach Suggests That Butyrophilins Have Ligands Beyond Gamma–Delta Receptors

**DOI:** 10.3390/ijms27020741

**Published:** 2026-01-11

**Authors:** Ludovic Marenco, Daniel Olive, Pierre Pontarotti

**Affiliations:** 1 Aix Marseille Aix Marseille Université, MEPHI, 13005 Marseille, France; ludovic.marenco@etu.univ-amu.fr; 2 IHU Méditerranée Infection, 13005 Marseille, France; 3 CNRS SNC5039, 13005 Marseille, France; 4 Team Immunity and Cancer, CRCM, Aix Marseille Université, CNRS, INSERM, Institut Paoli-Calmettes, 13005 Marseille, France; daniel.olive@inserm.fr

**Keywords:** butyrophilins, gamma-delta (γδ) T lymphocytes, immune system, squamates

## Abstract

Since γδ T cells are present in all jawed vertebrates, we wondered whether butyrophilins, proteins that play a key role in the activation of these cells, were also present in these organisms. Our analyses revealed the presence of genes encoding butyrophilins across all jawed vertebrates, including in squamates, a reptilian clade that is nonetheless reported in the literature to have lost γδ T cells. The conservation of butyrophilins in this group, despite the absence of their only known cellular partner, suggests that they may fulfill an alternative function, possibly through interaction with another ligand. Given their strong conservation across jawed vertebrates, it is reasonable to hypothesize that this alternative ligand may also be present in humans.

## 1. Introduction

The immune system is composed of a wide array of effector cells that defend the host against potential attacks and damage. T lymphocytes are among these effectors and can be divided into two classes based on their T-cell receptor (TCR): CD4 and CD8 T lymphocytes, which bear an αβ TCR, and γδ T lymphocytes, which bear a γδ TCR. The αβ T lymphocytes comprise approximately 90–99% of all T lymphocytes and play helper “CD4 helper” or defensive “CD8 cytotoxic” roles [1]. In contrast, γδ T lymphocytes are much less abundant, representing only about 1–10% of T lymphocytes, and play an important role in defense against viral and bacterial infections as well as in antitumor immunity. Moreover, unlike CD4 and CD8 T lymphocytes, which recognize peptides presented by MHC molecules, γδ T lymphocytes recognize antigens in their native form, including stress ligands, lipids, and nonclassical MHC I molecules and so, are not restricted by MHC presentation. One hallmark of these cells is their ability to recognize phosphoantigens (pAgs), which are intermediates of the isoprenoid biosynthetic pathway, through specific butyrophilins. These butyrophilins are expressed on the surface of many cell types and are related to the B7 costimulatory receptors CD80 and CD86 through strong structural homology, characterized by the presence of IgV and IgC2 immunoglobulin (Ig) domains [2,3]. These butyrophilins signal the accumulation of these metabolites during stress or infection and enable the activation of γδ T lymphocytes. Upon pAg accumulation, these metabolites are detected by the intracellular B30.2 domains of a butyrophilin dimer, triggering a conformational change that allows target cells to be recognized by γδ T lymphocytes and, in turn, promotes their activation [4]. Since γδ T lymphocytes infiltrate most human tumors, they are promising candidates for therapeutic approaches [5,6,7], making the study of butyrophilins highly relevant. However, despite their central role in γδ T lymphocyte activation, butyrophilins have been well described in mammals but remain largely unexplored in other jawed vertebrates. In addition, it has been reported that among squamates, a reptilian clade including snakes, lizards, and amphisbaenians, the species *Tiliqua rugosa* lacks γδ TCRs [8]. Our preliminary analyses in this species also indicate an absence of butyrophilins, allowing us to hypothesize a potential co-evolution between butyrophilins and γδ TCRs. To test this hypothesis, we first sought to determine whether the absence of γδ TCRs is a general feature of all squamates or a trait specific to *Tiliqua rugosa*. Once this was addressed, we then examined the conservation of butyrophilins across jawed vertebrates.

## 2. Results

### 2.1. Absence of γδ T Cells in All Squamates

**Figure 1:**
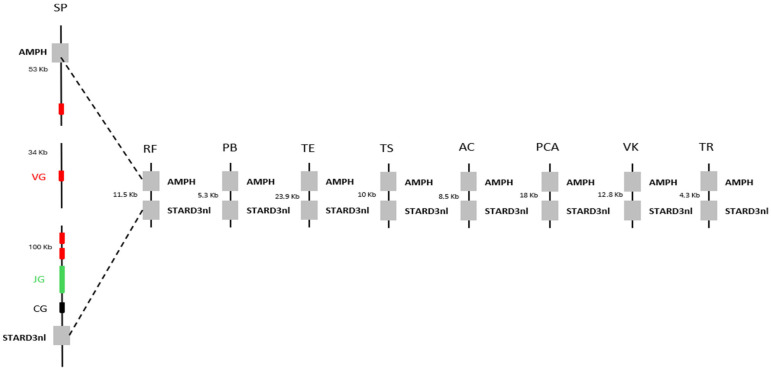
Decorrelation between the presence of T γδ cells and the conservation of butyrophilins in squamates. Observation of the deletion of T γδ cells through a comparative genomic analysis of the distance between the AMPH and STARD3NL genes, which flank the γ chain in *Sphenodon punctatus* (SP), the closest living relative of squamates. VG, JG, and CG correspond, respectively, to the Vγ chain, Jγ chain, and Cγ chain. The squamates included in this analysis are: *Rhineura floridana* (RF), *Python bivittatus* (PB), *Thamnophis elegans* (TE), *Tiliqua scincoides* (TS), *Anolis carolinensis* (AC), *Pituophis catenifer annectens* (PCA), *Varanus komodoensis* (VK), and *Tiliqua rugosa* (TR).

We first sought to determine whether the absence of γδ T cells was common to all squamates. To this end, we mapped the AMPH and STARD3NL anchor genes, which are known to flank the γ chain in many organisms [8], onto the genomes of various squamates. The distance between these anchors was then compared to the distance observed in the genome of *Sphenodon punctatus*, the closest living relative of squamates [9]. The distance between these anchors was found to be much smaller in squamates than in *Sphenodon punctatus*, with an approximately 200 kb deletion identified between the anchors (Figure 1). The δ chain was then searched across squamates using BlastP or TBlastn, based on the chicken (*Gallus gallus*) δ chain sequence, as the chicken is the best-studied organism among the closest relatives of squamates. However, due to the high sequence similarity between the β and δ chains, it was not possible to clearly assess the deletion of the δ chain. In fact, this approach indicated the presence of a δ variable chain but the absence of the δ constant chain in the majority of squamates. We therefore chose to represent the loss of γδ T cells in squamates solely by the absence of the γ chain. Together, these results suggest a widespread loss of γδ T cells across all squamates, rather than being restricted to *Tiliqua rugosa*.

### 2.2. Conservation of Butyrophilins Across Jawed Vertebrates

**Figure 2:**
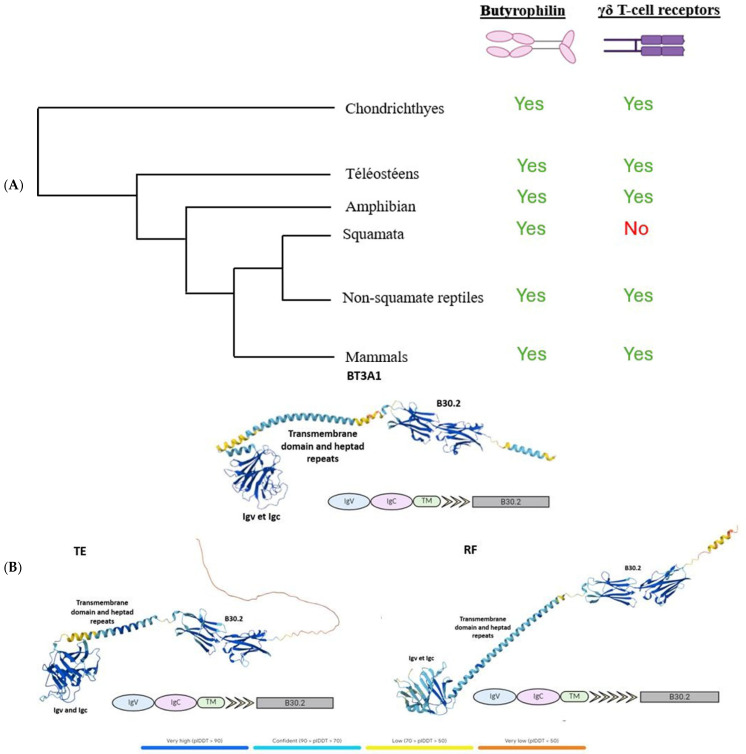
Study of the functionality of butyrophilins in squamates. (**A**), Simplified phylogeny of jawed vertebrates, indicating the presence or absence of butyrophilins and T γδ receptors across different species. (**B**), Comparison of the predicted structures of butyrophilins from the squamates *Thamnophis elegans* (TE) and *Rhineura floridana* (RF) with that of the human BTN3A1, which has been previously characterized. The identified domains are indicated on each butyrophilin.

γδ T cells are known to be expressed exclusively in jawed vertebrates. Butyrophilins, meanwhile, have only been described as interacting with these cells in humans, mice, and alpacas [10], while their characteristics remain largely unexplored in other jawed vertebrates. After demonstrating the widespread absence of γδ T lymphocytes across all squamates, we sought to investigate the conservation of butyrophilins in jawed vertebrates in order to assess the potential co-evolution between butyrophilins and the γδ TCR. To this end, we used the human butyrophilin BTN3A1 sequence, known for its role in phosphoantigen recognition and subsequent activation of γδ T lymphocytes [11,12,13], as a query to identify butyrophilins across a range of species, employing BLASTp or tBLASTn for incomplete genome assemblies (see Section 4). These analyses revealed the presence of butyrophilins in all species tested, including squamates, despite their lack of γδ TCRs (Figure 2A). In light of these results, we wondered about the functionality of the butyrophilins identified in squamates and whether these might instead be artifacts or ancestral pseudogenes retained following the loss of γδ T lymphocytes. To investigate this, we used the AlphaFold prediction tool (version DB v6, accessed on 21 May 2025) to model the structures of butyrophilins from two squamates, *Thamnophis elegans* (TE) and *Rhineura floridana* (RF), and compared them to the human butyrophilin BT3A1. Structurally, the squamate butyrophilins exhibited a high degree of similarity to human BT3A1, including readily identifiable IgV, IgC, and B30.2 domains, a transmembrane domain, and a variable number of heptad repeats (Figure 2B). The only notable difference was in the number of heptad motifs downstream of the transmembrane domain (Figure 2B). These motifs, primarily structural, are frequently involved in the stabilization of coiled-coil helices [14]. Since the IgV, IgC, and B30.2 domains are conserved, this structural difference is unlikely to affect the functional role of the butyrophilins. Predicted structures for butyrophilins from other squamates mentioned in this article are presented in Figure S1. The finding that butyrophilins are conserved across all jawed vertebrates, combined with the fact that γδ T lymphocytes occur exclusively in this group, suggests that butyrophilins and γδ T lymphocytes emerged simultaneously in the common ancestor of jawed vertebrates, highlighting the critical role of butyrophilins for these lymphocytes. The construction of a phylogeny of butyrophilins across selected organisms further clarified the relationships between these molecules, revealing that butyrophilins are class-specific and have undergone numerous lineage-specific duplication events (Figure S2).

Most importantly, the discovery of butyrophilins highly similar to the human BTN3A1 and apparently functional in squamates despite the absence of γδ TCRs contradicts the hypothesis of a co-evolution between butyrophilins and γδ TCRs. Considering that squamates emerged roughly 240 million years ago, and that the oldest known fossils date to approximately 170 million years ago, it is highly improbable that butyrophilins would have been conserved for such a long period without any functional role or binding partner. These results thus point to the existence of alternative ligands beyond the γδ TCR that can interact with butyrophilins.

### 2.3. Chromosomal Proximity Between Butyrophilin Genes and MHC Class I Genes in Most Jawed Vertebrates

**Figure 3:**
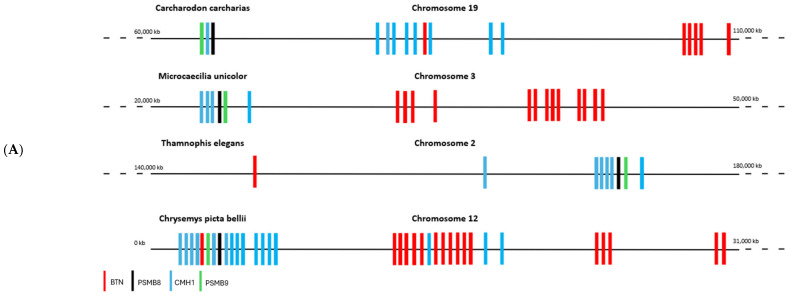

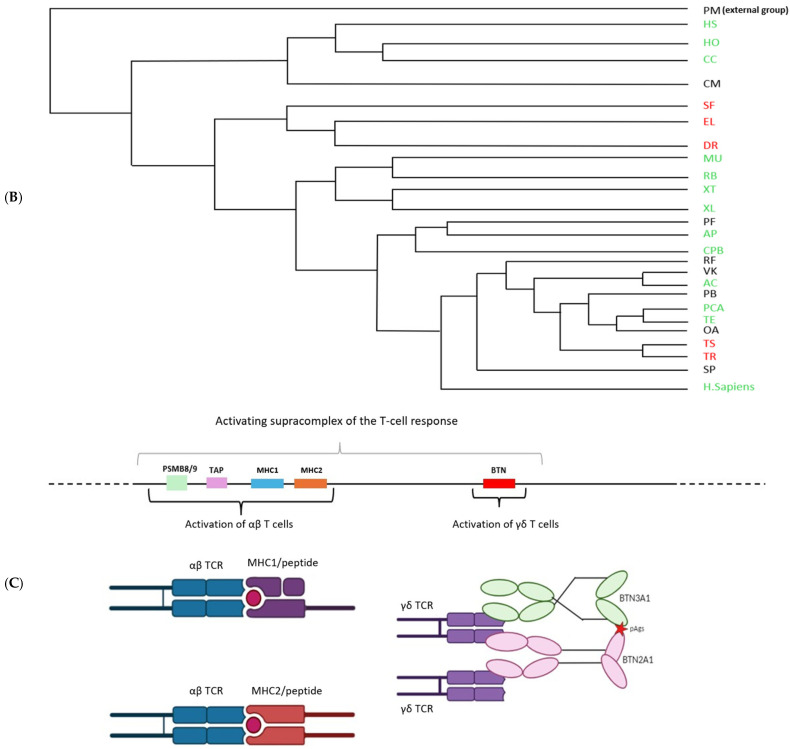
Chromosomal mapping of the genes encoding butyrophilins and hypothesis of a T-cell activating supracomplex. (**A**), Localization of the genes encoding butyrophilins and class I MHC molecules on the genome of various jawed vertebrates. The genes encoding PSMB8 and PSMB9 were also mapped to confirm the correct positioning of the class I MHC genes. (**B**), Phylogenetic tree illustrating the relationships among butyrophilins in jawed vertebrates. Organisms indicated in green possess genes encoding butyrophilins located near class I MHC genes. Those in red represent these genes on separate chromosomes. Organisms in black correspond to cases for which chromosomal localization data are not available. Abbreviations of the Latin names and the classification of the different phyla are presented in Section 4. (**C**), Reconstruction of the ancestral activating supracomplex involved in the activation of αβ and γδ T cells.

The literature has shown that butyrophilins capable of interacting with γδ T cells are located within the same locus as MHC class I and class II molecules, which enable the activation of αβ T cells [15]. Only certain butyrophilins do not belong to this locus; this is the case for BTNL9, BTNL8, and BTNL3 in humans [15]. After demonstrating that butyrophilins are conserved across all jawed vertebrates, we then sought to determine whether they are also located within the same locus as MHC class I and class II molecules. To do this, the sequences of MHC class I molecules from the same organisms studied in the first part, in particular those shown in Figure 1, were identified using the human sequence with the BLASTp tool (version BLAST+ 2.16.0, accessed on 2 April 2025), then located on the corresponding genomes using tBLASTn. Since MHC class I molecules are relatively divergent between species (≈30% identity), we retrieved the sequences of the proteasome subunits PSMB8 and PSMB9, which are known to flank MHC class I genes and show low interspecies divergence (≈70% identity), in order to confirm the correct positioning of the MHC class I genes on the genomes. In the same way, the butyrophilin sequences obtained in the first part were also mapped onto the genome. These analyses enabled us to identify numerous organisms in which the butyrophilins are located on the same chromosome as MHC class I molecules, at varying distances from one another (Figure 3A). This is notably the case for squamates such as *Thamnophis elegans*, *Pituophis catenifer annectens*, and *Anolis carolinensis*; for amphibians such as *Microcaecilia unicolor*, *Rhinatrema bivittatum*, *Xenopus tropicalis*, and *Xenopus laevis*; for non-squamate reptiles such as *Chrysemys picta bellii*; and for cartilaginous fishes such as *Hemiscyllium ocellatum*, *Carcharodon carcharias*, and *Hypanus sabinus* (Figure 3B). However, in these organisms, it is still possible to detect some butyrophilins on other, distinct chromosomes, as is the case in humans [15]. There are nevertheless certain exceptions in which all the butyrophilins are located on a chromosome distinct from that of the MHC class I genes. This is the case, for example, in the squamates *Rhineura floridana*, *Tiliqua scincoides*, and *Ophiophagus hannah*; in the reptile *Patagioenas fasciata*; and in the chondrichthyan *Callorhinchus milii* (Figure 3B). In teleosts (bony fish), no species displays butyrophilins located on the same chromosome as MHC class I genes. In contrast to the other vertebrate classes, MHC class II genes are also located on a different chromosome [16,17]. This separation of these elements onto different chromosomes in teleosts can be explained by the number of genome duplications they have undergone [18,19], which has resulted in extensive chromosomal rearrangements. The presence of butyrophilin genes near MHC genes in the majority of jawed vertebrates suggests an ancient physical association between these two gene families. This proximity may also reflect a functional link, insofar as jawed vertebrates possess both αβ and γδ T cells: MHC molecules participate in the activation of αβ T cells, while butyrophilins play an essential role in the activation of γδ T cells. These concepts, together with the hypothesis that butyrophilins emerged at the same time as γδ T cells in their common ancestor, suggest that this ancestor possessed a supra-genetic complex located on the same chromosome and involved in the activation of both αβ and γδ T cells (Figure 3C). It therefore appears that a physical and functional link between the genes encoding MHC class I molecules and those encoding butyrophilins represented a plesiomorphic character in the common ancestor of jawed vertebrates. However, this character was subsequently lost independently in certain organisms or lineages, leading to an apomorphic state marked by the separation of these genes onto distinct chromosomes.

## 3. Discussion

Butyrophilins are primarily known for their ability to interact with γδ T lymphocytes, leading to their activation. This capacity to stimulate rare but critically important immune cells in antitumor responses has made these proteins promising therapeutic candidates. Despite this, butyrophilins remain largely understudied, and their specific interactions have been characterized only in a handful of mammals, most notably in humans, mice, and alpacas. This paucity of data motivated our interest in butyrophilins, and more generally in their evolution relative to the γδ TCR. Our initial hypothesis was that butyrophilins and γδ TCRs might have co-evolved. To test this, we first examined the conservation of γδ TCR genes across jawed vertebrates, focusing especially on squamates, given that *Tiliqua rugosa*, a representative of this clade, has been reported to lack these receptors. Our analysis revealed a generalized absence of γδ T lymphocytes across all squamates, and not merely in *T. rugosa*.

Following this discovery, we investigated the conservation of butyrophilins across jawed vertebrates to assess the validity of the co-evolution hypothesis. These analyses confirmed the presence of butyrophilins in every species tested, including the squamates that lack a γδ TCR. The predicted structures of butyrophilins in squamates appear functional, suggesting that these are not ancestral artifacts or pseudogenes. The strong conservation of butyrophilins across jawed vertebrates, combined with their expression being limited, as is the case for γδ T lymphocytes, to this group [20,21], initially led us to hypothesize that both evolved simultaneously in their common ancestor. However, the fact that butyrophilins have been highly conserved across jawed vertebrates, despite their absence of association with γδ T lymphocytes in squamates, contradicts the notion of their co-evolution. The loss of γδ T lymphocytes in squamates appears to have occurred after their divergence from the tuatara (sphenodon punctatus), approximately 240 million years ago. This timeline is supported by fossil evidence placing the oldest squamate ancestor at roughly 168 million years ago [22]. The persistence of genes encoding butyrophilins for such an extended period, despite the absence of γδ T lymphocytes, strongly suggests that these molecules have assumed an alternative role, likely through interactions with a distinct ligand. This is further corroborated by recent work highlighting a novel function for butyrophilins. In particular, engagement of BTN2A1 expressed on M2-polarized macrophages and tumor-associated macrophages (TAMs) via a specific antibody promotes their reprogramming into M1-polarized macrophages through the SYK and MAPK signaling pathways [23]. This finding, which illustrates an alternate role for butyrophilins, supports the hypothesis that ligands other than the γδ TCR may interact with these molecules, although such ligands have yet to be identified. It is very likely that this ability to bind an alternative partner has enabled the conservation of butyrophilins in squamates despite the loss of γδ T lymphocytes.

Considering the significant therapeutic potential of reprogramming TAMs to an M1-like phenotype within the tumor microenvironment, it would be highly relevant to identify this ligand in humans. To this end, a proteomics-based approach, such as a pull-down assay with a tagged, recombinant BTN2A1 protein could be implemented. After expressing and purifying the tagged BTN2A1, it would be immobilized on a solid matrix and incubated with cellular lysates, followed by elution and identification of bound proteins by mass spectrometry. Should such a ligand be identified in humans, it would be equally pertinent to investigate its presence in squamates to assess whether the same or an alternative binding partner is used in these species. In the latter case, this would point to the existence of a distinct ligand that can interact with butyrophilins. Given the strong conservation of butyrophilins across jawed vertebrates, the discovery of an alternate binding partner in squamates would imply that the same ligand may also be present in other jawed vertebrates, including humans. This putative ligand could interact with butyrophilins already known for their role in γδ T lymphocyte activation or with other butyrophilins for which ligands have yet to be identified [15], thereby fulfilling a distinct functional role, akin to BTN2A1 in M2-polarized and TAMs [23].

## 4. Materials and Methods

### 4.1. Organisms Analyzed

The analyses were performed using genomic sequences from twenty-six species representative of jawed vertebrate diversity, as well as the sea lamprey, used as a representative of cyclostomes, an outgroup to jawed vertebrates. The selected species are: *Petromyzon marinus* (PM), *Patagioenas fasciata* (PF), *Anas platyrhynchos* (AP), *Rhineura floridana* (RF), *Python bivittatus* (PB), *Tiliqua scincoides* (TS), *Tiliqua rugosa* (TR), *Varanus komodoensis* (VK), *Pituophis catenifer annectens* (PCA), *Thamnophis elegans* (TE), *Anolis carolinensis* (AC), *Ophiophagus hannah* (OA), *Sphenodon punctatus* (SP), *Chrysemys picta bellii* (CPB), *Microcaecilia unicolor* (MU), *Rhinatrema bivittatum* (RB), *Xenopus tropicalis* (XT), *Xenopus laevis* (XL), *Scleropages formosus* (SF), *Esox lucius* (EL), *Danio rerio* (DR), *Hemiscyllium ocellatum* (HO), *Hypanus sabinus* (HS), *Carcharodon carcharias* (CC), *Callorhinchus milii* (CM), *Homo sapiens*.

### 4.2. Phylogenetic Classification

(PM) was used as the outgroup in the phylogeny because it belongs to the cyclostomes, a group of jawless vertebrates. (HO), (HS), (CC), and (CM) belong to Chondrichthyes (cartilaginous fishes). (SF), (EL), and (DR) belong to Osteichthyes (bony fishes). (MU), (RB), (XT), and (XL) are amphibians. (PF), (AP), and (CPB) belong to non-squamate reptiles (birds and turtles). (RF), (PB), (TS), (VK), (PCA), (TE), (AC), (OA), and (TR) are squamates, a clade of reptiles. (SP) belongs to Rhynchocephalians, a group of reptiles distinct from squamates, for which it is the only extant representative.

### 4.3. Search for Butyrophilins and Other Proteins

Protein sequences of interest were retrieved from the human database UniProt. These sequences were then used as queries to identify orthologs in the genomes of the target organisms. For complete genomes, the search was performed using the BLASTp tool (version BLAST+ 2.16.0, accessed on 2 April 2025) on the NCBI platform, while for incomplete genomes, the tBLASTn tool (version BLAST+ 2.16.0, accessed on 2 April 2025) was used to query translated nucleotide sequences. Default parameters of the NCBI tools were applied for all analyses. (https://blast.ncbi.nlm.nih.gov/Blast.cgi?PROGRAM=blastn&PAGE_TYPE=BlastSearch&LINK_LOC=blasthome (accessed on 2 April 2025)).

### 4.4. Chromosomal Localization of Proteins

The retrieved protein sequences were mapped onto the genomes of the species of interest using the tBLASTn tool (version BLAST+ 2.16.0, accessed on 2 April 2025). Default parameters of the tool were used for all analyses.

### 4.5. Phylogeny

The phylogenetic tree representing the overall phylogeny of the species was constructed using the Mesquite software (version 4.02), based on a NEWICK format tree provided by the “taxonomy” tool from the NCBI platform (https://www.ncbi.nlm.nih.gov/Taxonomy/CommonTree/wwwcmt.cgi, accessed on 13 May 2025). The phylogeny of butyrophilins from the different organisms (Figure S2) was performed using the MEGA software (https://www.megasoftware.net/, accessed on 7 April 2025, software version 12.0.10). The imported sequences were aligned using the MUSCLE algorithm, employing a clustering strategy based on the Neighbor- Joining method, which is well-suited for distance-based phylogenetic analyses, especially when sequences are divergent. Sequences that were too long or poorly aligned were removed to minimize alignment artifacts that could affect phylogenetic reconstruction. The phylogenetic tree was built by applying a complete deletion of positions containing gaps, and robustness was assessed by bootstrap analysis with 500 replicates.

### 4.6. Figures

The depiction of the interactions between TCR αβ and γδ with MHC molecules and butyrophilins, respectively, was created using the BioRender tool (https://www.biorender.com/, accessed on 10 April 2025). The structural representations of butyrophilins (Figure 2B and Figure S1) were generated using the AlphaFold tool (version: DB v6). The butyrophilins of squamates are predicted structures, whereas the human butyrophilin has already been characterized.

### 4.7. Dating

The TimeTree tool (version 5.1) was used to estimate the divergence times between different species. In particular, it was used to date the split between the tuatara (sphenodon punctatus) and the squamates.

## 5. Conclusions

The conservation of butyrophilin genes in squamate taxa, in which γδ T cells are reported to be absent, suggests that butyrophilins may fulfill an alternative function, possibly through interaction with a ligand other than the γδ T-cell receptor.

## Data Availability

The original contributions presented in this study are included in the article. Further inquiries can be directed to the corresponding author, Pierre Pontarotti.

## Supplementary Materials

The following supporting information can be downloaded at: https://www.mdpi.com/article/10.3390/ijms27020741/s1.
