# Supplementary material for: The Phylogenomic Approach Suggests That Butyrophilins Have Ligands Beyond Gamma–Delta Receptors
Source: Int J Mol Sci. 2026 Jan 11;27(2):741. doi: 10.3390/ijms27020741 (PMC12841164; doi:10.3390/ijms27020741)
Supplement: Supplementary file 1 [file ijms-27-00741-s001.zip › ijms-3905618-supplementary.pdf]

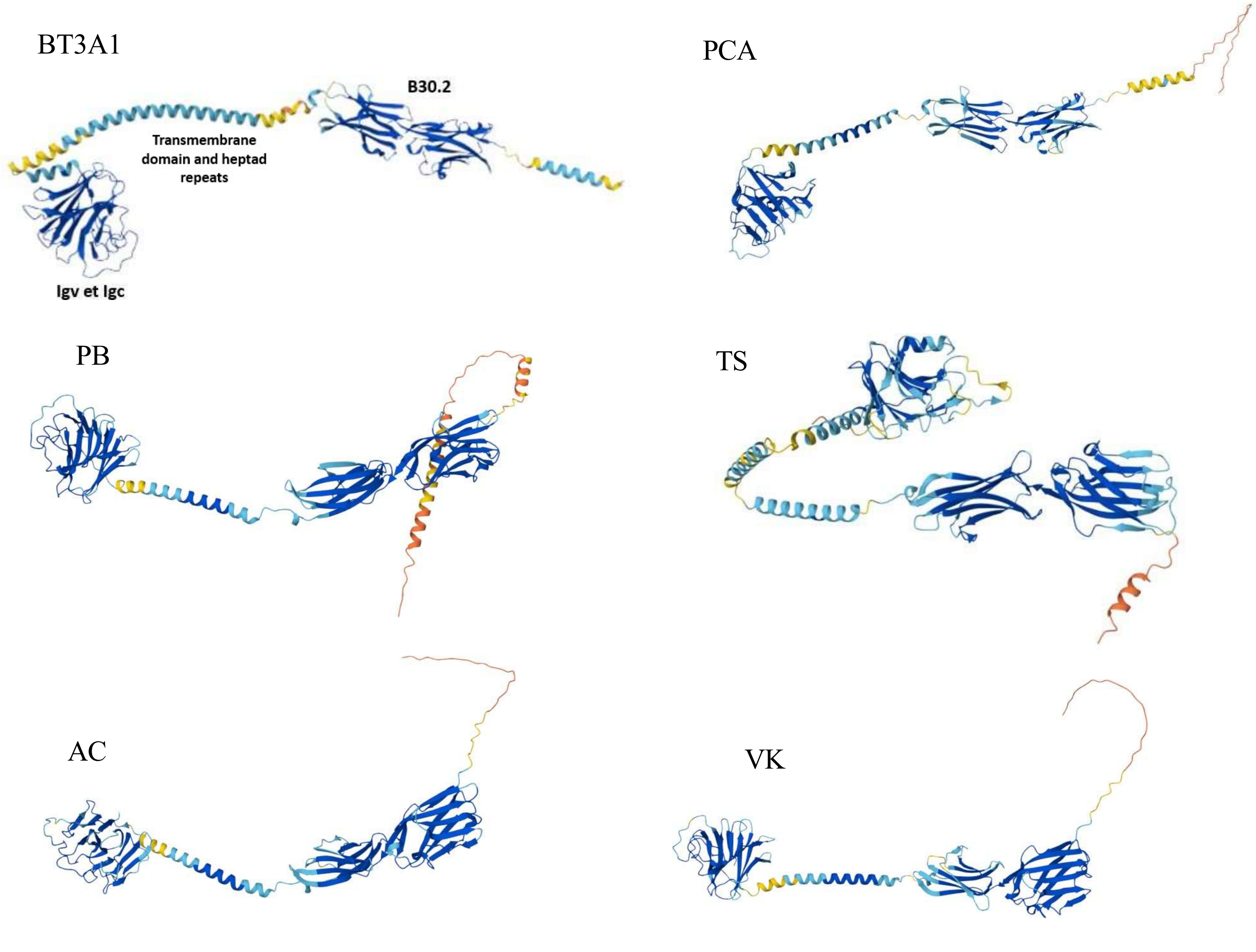

Figure S1 | Structural prediction of butyrophilins in the squamates analyzed.
Predicted structures of butyrophilins in the examined squamate species, with the exception of *Ophiophagus hannah*, for which the prediction was of low quality. The human butyrophilin BT3A1 was also included for comparative purposes. The Latin name abbreviations are presented in the "Materials and Methods" section.

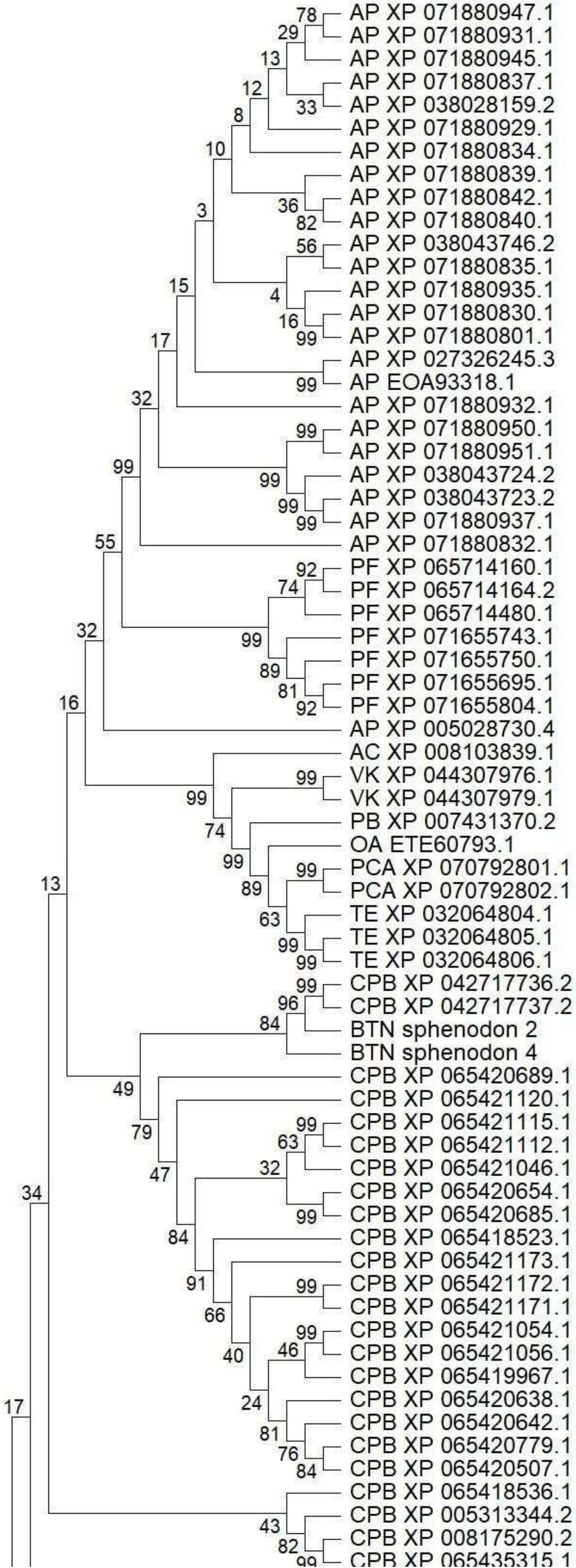
AP XP 071880947.1
AP XP 071880931.1
AP XP 071880945.1
AP XP 071880837.1
AP XP 038028159.2
AP XP 071880929.1
AP XP 071880834.1
AP XP 071880839.1
AP XP 071880842.1
AP XP 071880840.1
AP XP 038043746.2
AP XP 071880835.1
AP XP 071880935.1
AP XP 071880830.1
AP XP 071880801.1
AP XP 027326245.3
AP EOA93318.1
AP XP 071880932.1
AP XP 071880950.1
AP XP 071880951.1
AP XP 038043724.2
AP XP 038043723.2
AP XP 071880937.1
AP XP 071880832.1
PF XP 065714160.1
PF XP 065714164.2
PF XP 065714480.1
PF XP 071655743.1
PF XP 071655750.1
PF XP 071655695.1
PF XP 071655804.1
AP XP 005028730.4
AC XP 008103839.1
VK XP 044307976.1
VK XP 044307979.1
PB XP 007431370.2
OA ETE60793.1
PCA XP 070792801.1
PCA XP 070792802.1
TE XP 032064804.1
TE XP 032064805.1
TE XP 032064806.1
CPB XP 042717736.2
CPB XP 042717737.2
BTN sphenodon 2
BTN sphenodon 4
CPB XP 065420689.1
CPB XP 065421120.1
CPB XP 065421115.1
CPB XP 065421112.1
CPB XP 065421046.1
CPB XP 065420654.1
CPB XP 065420685.1
CPB XP 065418523.1
CPB XP 065421173.1
CPB XP 065421172.1
CPB XP 065421171.1
CPB XP 065421054.1
CPB XP 065421056.1
CPB XP 065419967.1
CPB XP 065420638.1
CPB XP 065420642.1
CPB XP 065420779.1
CPB XP 065420507.1
CPB XP 065418536.1
CPB XP 005313344.2
CPB XP 008175290.2

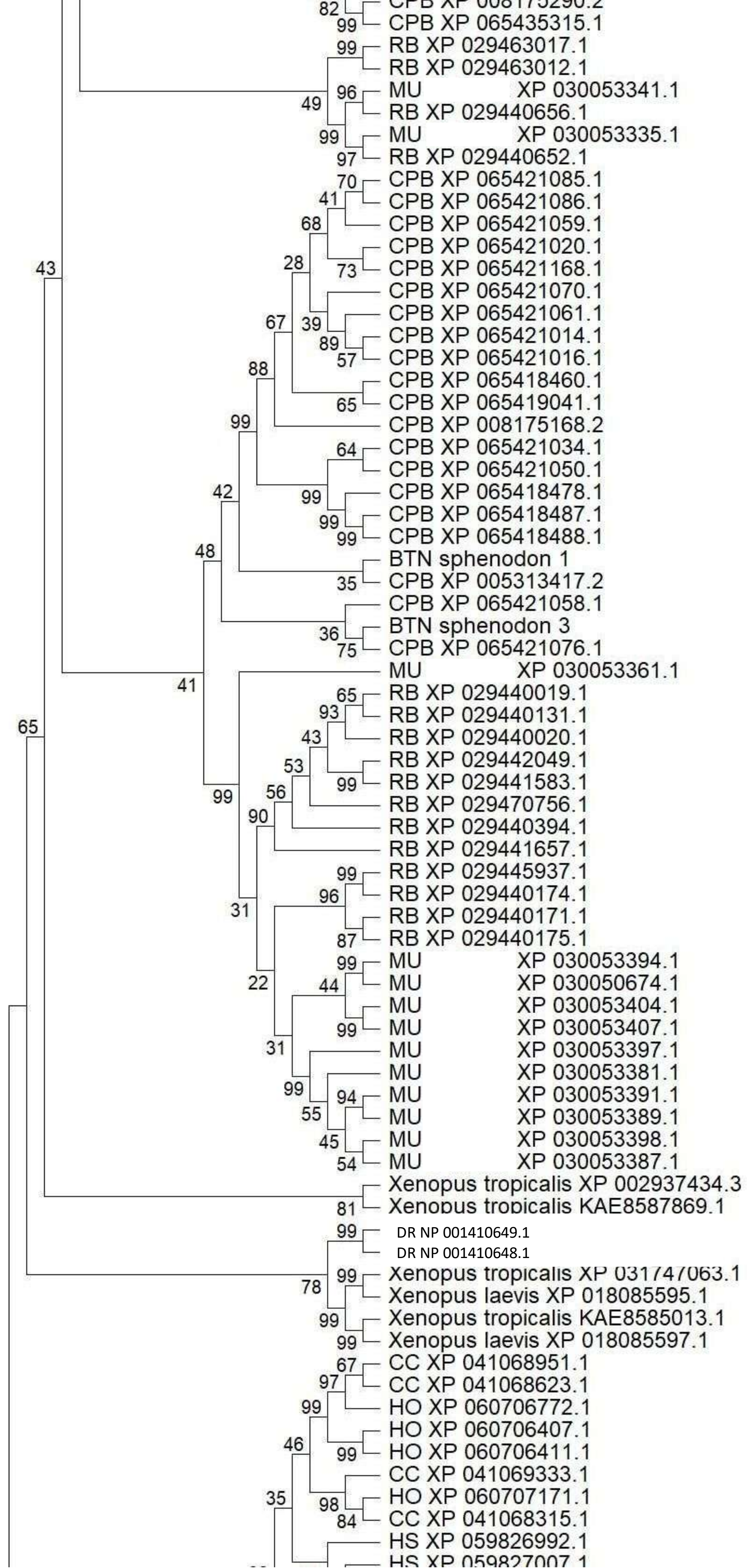
CPB XP 065435315.1
RB XP 029463017.1
RB XP 029463012.1
MU XP 030053341.1
RB XP 029440656.1
MU XP 030053335.1
RB XP 029440652.1
CPB XP 065421085.1
CPB XP 065421086.1
CPB XP 065421059.1
CPB XP 065421020.1
CPB XP 065421168.1
CPB XP 065421070.1
CPB XP 065421061.1
CPB XP 065421014.1
CPB XP 065421016.1
CPB XP 065418460.1
CPB XP 065419041.1
CPB XP 008175168.2
CPB XP 065421034.1
CPB XP 065421050.1
CPB XP 065418478.1
CPB XP 065418487.1
CPB XP 065418488.1
BTN sphenodon 1
CPB XP 005313417.2
CPB XP 065421058.1
BTN sphenodon 3
CPB XP 065421076.1
MU XP 030053361.1
RB XP 029440019.1
RB XP 029440131.1
RB XP 029440020.1
RB XP 029442049.1
RB XP 029441583.1
RB XP 029470756.1
RB XP 029440394.1
RB XP 029441657.1
RB XP 029445937.1
RB XP 029440174.1
RB XP 029440171.1
RB XP 029440175.1
MU XP 030053394.1
MU XP 030050674.1
MU XP 030053404.1
MU XP 030053407.1
MU XP 030053397.1
MU XP 030053381.1
MU XP 030053391.1
MU XP 030053389.1
MU XP 030053398.1
MU XP 030053387.1
Xenopus tropicalis XP 002937434.3
Xenopus tropicalis KAE8587869.1
DR NP 001410649.1
DR NP 001410648.1
Xenopus tropicalis XP 031747063.1
Xenopus laevis XP 018085595.1
Xenopus tropicalis KAE8585013.1
Xenopus laevis XP 018085597.1
CC XP 041068951.1
CC XP 041068623.1
HO XP 060706772.1
HO XP 060706407.1
HO XP 060706411.1
CC XP 041069333.1
HO XP 060707171.1
CC XP 041068315.1
HS XP 059826992.1

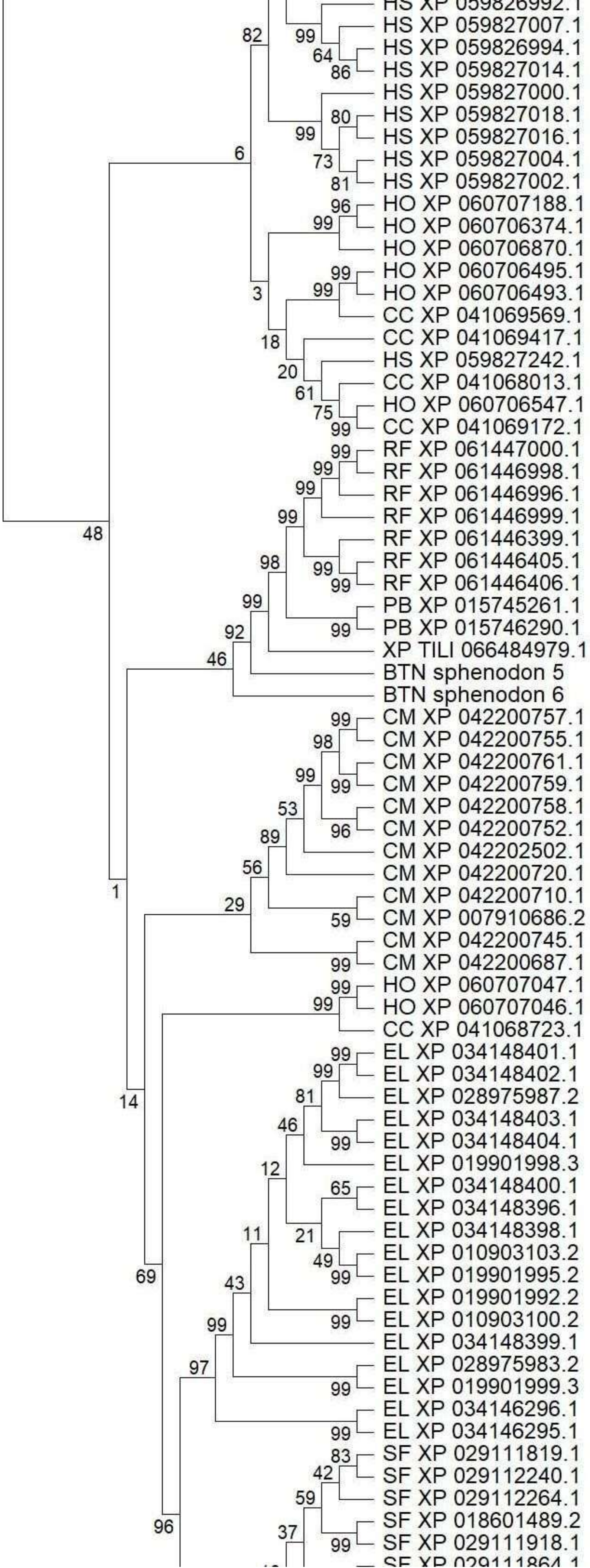
HS XP 059826992.1
HS XP 059827007.1
HS XP 059826994.1
HS XP 059827014.1
HS XP 059827000.1
HS XP 059827018.1
HS XP 059827016.1
HS XP 059827004.1
HS XP 059827002.1
HO XP 060707188.1
HO XP 060706374.1
HO XP 060706870.1
HO XP 060706495.1
HO XP 060706493.1
CC XP 041069569.1
CC XP 041069417.1
HS XP 059827242.1
CC XP 041068013.1
HO XP 060706547.1
CC XP 041069172.1
RF XP 061447000.1
RF XP 061446998.1
RF XP 061446996.1
RF XP 061446999.1
RF XP 061446399.1
RF XP 061446405.1
RF XP 061446406.1
PB XP 015745261.1
PB XP 015746290.1
XP TILI 066484979.1
BTN sphenodon 5
BTN sphenodon 6
CM XP 042200757.1
CM XP 042200755.1
CM XP 042200761.1
CM XP 042200759.1
CM XP 042200758.1
CM XP 042200752.1
CM XP 042202502.1
CM XP 042200720.1
CM XP 042200710.1
CM XP 007910686.2
CM XP 042200745.1
CM XP 042200687.1
HO XP 060707047.1
HO XP 060707046.1
CC XP 041068723.1
EL XP 034148401.1
EL XP 034148402.1
EL XP 028975987.2
EL XP 034148403.1
EL XP 034148404.1
EL XP 019901998.3
EL XP 034148400.1
EL XP 034148396.1
EL XP 034148398.1
EL XP 010903103.2
EL XP 019901995.2
EL XP 019901992.2
EL XP 010903100.2
EL XP 034148399.1
EL XP 028975983.2
EL XP 019901999.3
EL XP 034146296.1
EL XP 034146295.1
SF XP 029111819.1
SF XP 029112240.1
SF XP 029112264.1
SF XP 018601489.2
SF XP 029111918.1

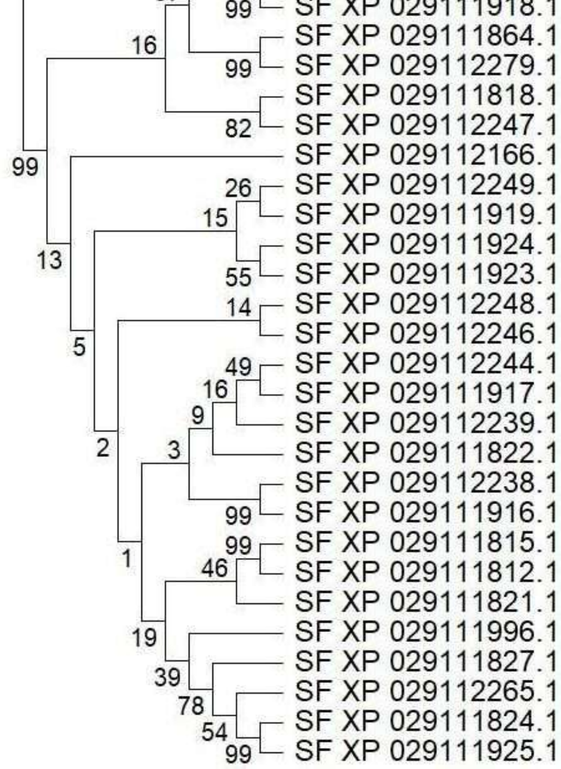

Figure S2 | Phylogeny of butyrophilins in jawed vertebrates.
Phylogenetic tree generated using MEGA, illustrating the evolutionary relationships among butyrophilins in jawed vertebrates. Bootstrap values below 70 are not considered statistically significant and should be interpreted with caution. The Latin name abbreviations are detailed in the "Materials and Methods" section, except for **TILI**, which stands for *Tiliqua scincoides*. The butyrophilins are accompanied by their corresponding accession numbers, except for that of *Sphenodon punctatus*, whose sequence was manually reconstructed from genome fragments identified using tBLASTn, as the protein has not yet been sequenced.
